# Increased TLR4 Expression and Downstream Cytokine Production in Immunosuppressed Adults Compared to Non-Immunosuppressed Adults

**DOI:** 10.1371/journal.pone.0011343

**Published:** 2010-06-28

**Authors:** Dana W. Dunne, Albert Shaw, Linda K. Bockenstedt, Heather G. Allore, Shu Chen, Stephen E. Malawista, Lin Leng, Yuka Mizue, Marta Piecychna, Lin Zhang, Virginia Towle, Richard Bucala, Ruth R. Montgomery, Erol Fikrig

**Affiliations:** 1 Department of Internal Medicine, Yale University School of Medicine, New Haven, Connecticut, United States of America; 2 Sapporo Immuno Diagnostic Laboratory, Sapporo, Japan; 3 Howard Hughes Medical Institute, Chevy Chase, Maryland, United States of America; New York University, United States of America

## Abstract

**Background:**

An increasing number of patients have medical conditions with altered host immunity or that require immunosuppressive medications. While immunosuppression is associated with increased risk of infection, the precise effect of immunosuppression on innate immunity is not well understood. We studied monocyte Toll-like receptor (TLR) expression and cytokine production in 137 patients with autoimmune diseases who were maintained on immunosuppressive medications and 419 non-immunosuppressed individuals.

**Methodology/Principal Findings:**

Human peripheral blood monocytes were assessed for surface expression of TLRs 1, 2, and 4. After incubation with TLR agonists, *in vitro* production of the cytokines IL-8, TNFα, and MIF were measured by ELISA as a measure of TLR signaling efficiency and downstream effector responsiveness. Immunosuppressed patients had significantly higher TLR4 surface expression when compared to non-immunosuppressed adults (TLR4 %-positive 70.12±2.28 vs. 61.72±2.05, p = 0.0008). IL-8 and TNF-α baseline levels did not differ, but were significantly higher in the autoimmune disease group following TLR stimulation. By contrast, baseline MIF levels were elevated in monocytes from immunosuppressed individuals. By multivariable analyses, IL-8 and TNFα, but not MIF levels, were associated with the diagnosis of an underlying autoimmune disease. However, only MIF levels were significantly associated with the use of immunosuppressive medications.

**Conclusions/Significance:**

Our results reveal that an enhanced innate immune response is a feature of patients with autoimmune diseases treated with immunosuppressive agents. The increased risk for infection evident in this patient group may reflect a dysregulation rather than a simple suppression of innate immunity.

## Introduction

The past 10 years have seen an exponential growth in our understanding of the importance of Toll-like receptors (TLRs) in innate immunity. TLRs are a class of highly conserved pattern recognition receptors found in metazoan species that respond to conserved molecular patterns (also referred to as pathogen-associated molecular patterns or PAMPs) common to microbial pathogens [1], [2]. The 10 currently described human TLRs vary in their expression among immune cell types and in their recognition of microbial molecules [3]-[5]. TLRs 1, 2, 4, 5, and 6 are expressed on the cell surface and largely recognize bacterial and fungal PAMPs whereas TLRs 3, 7, 8, and 9 are predominantly found in intracellular sites and recognize viral and non-viral nucleic acids. This latter category of TLRs also may allow the host immune cells to undergo activation by endogenous nucleic acids thereby contributing to the pathogenesis of autoimmunity [6].

A potential role for TLR responses to self ligands in autoimmunity is emerging. Recent studies have demonstrated that endogenous nucleic acids may activate plasmacytoid dendritic cells (pDC) via TLR7 and TLR9, leading to the production of Type I interferons (INFα/β) [7], that may drive many of the clinical features of systemic lupus erythematosis (SLE) [8]. Experimental models of SLE using TLR7 and/or TLR9-deficient mouse strains have further clarified the role that these receptors play in the production of autoantibodies and in the development of immunopathology (reviewed in [9], [10]. Recently, the heat shock proteins (HSP) 96 and HSP22, ligands for TLR2 and 4, respectively, have been reported to play a role in the development of or exacerbation of rheumatoid arthritis [11], [12]. Whether alterations in TLR-mediated immune responses to foreign ligands such as PAMPs contribute to the increased susceptibility to infections seen in affected patients is unclear. In the present report, we sought to characterize the initial innate immune response in human subjects with autoimmune diseases receiving immunosuppressive therapy by evaluating monocyte TLR surface expression and innate cytokine production. We were able to demonstrate significant differences in elements of innate immunity in this special patient population.

## Results

### TLR expression in immunosuppressed adults

To better understand the impact of broadly defined immunosuppression on human TLR function, we enrolled 137 immunosuppressed adults and 419 non-immunosuppressed adults over the age of 21 (Table 1). There were a significantly higher proportion of middle-aged adults and women in the immunosuppressed group compared to the non-immunosuppressed group. Of the 137 immunosuppressed adults enrolled, 87% had a single disease requiring immunosuppression with the remaining 13% having more than one autoimmune disease (Table 2). The most common singularly occurring autoimmune disease in our sample was RA (46%). Other diseases for which subjects were receiving immunosuppressants are listed in Table 2. The majority of the immunosuppressed adults were on non-biologic medications alone (59%) (Table 2).

**Table 1:** Characteristics of subject cohorts.

	Non-immunosuppressed (n = 419)	Immunosuppressed (n = 137)	P value†
**Age, year**			
<40 years	148 (35.3%)	43 (31.4%)	---
40-59 years	68 (16.2%)	57 (41.6%)	0.04
>60 years	203(48.5%)	37 (27.0%)	---
Gender, female	248 (59.2%)	105 (76.6%)	0.0002
**Race**			
Caucasian (%)	352 (84.0%)	104 (75.9%)	0.039

† P values are based on X2 for categorical characteristics.

**Table 2:** Diseases and medications of enrolled immunosuppressed adults (N =  137).

Diagnosis	Number (%)
**Total Rheumatoid Arthritis (RA)**	74 (54.0%)
RA alone	63 (46.0%)
RA plus other	11 (8.0%)
Total Systemic lupus erythematosis (SLE)	21 (15.3%)
SLE alone	10 (7.3%)
SLE plus other	11(8.0%)
Other *alone	50 (36.5%)
**Medications**	**Number (%)**
Biologics #	22 (16.1%)
Non-biologics §	81 (59.1%)
Both	33 (24.1%)

*other- Ankylosing spondylitis (N = 2), antisynthetase syndrome (1), asthma (2), autoimmune hepatitis (1),Churgg-Strauss(1), Crohn's disease (1), dermatomyositis (1), fibromyalgia (1), hypogammaglobulinemia (1), inflammatory/reactive arthritis (4), mixed connective tissue disorder (2), multiple sclerosis (4), myositis (3), optic neuritis (1), polymyalgia rheumatica (4), psoriasis (3), psoriatic arthritis (5), sarcoidosis (3), scleroderma (1), Sjogren's syndrome (1), spondylarthrosis (1), Still's disease(2), thyroiditis (1), Wegener's granulomatosis (1).

#biologics- etanercept, adalimumab, infliximab, anakinra, abatacept, natalizumab.

§ non-biologics- azothioprine, cyclophosphamide, hydroxychloroquine, leflunomide, methotrexate, mycophenolate, prednisone.

**Figure 1:**
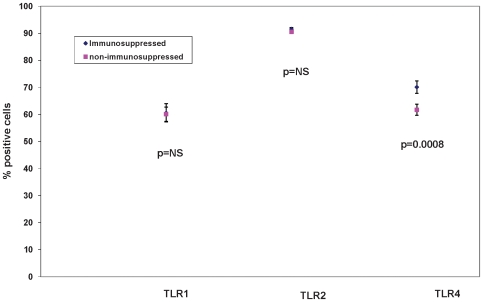
Surface expression of TLRs 1, 2 and 4 in Immunosuppressed adults compared to non-immunosuppressed adults. TLR surface expression is represented as percentage of CD4-dim cells stained with antibodies to TLR 1, 2, or 4 as assessed by flow cytometery. No statistical difference is seen in TLR 1 or 2 surface expressions in immunosuppressed adults compared to non-immunosuppressed adults. TLR4 surface expression was significantly increased in immunosuppressed adults (70.12±2.28 versus 61.72±2.05, p = 0.0008). NS =  p>0.05; values indicate least squares means from a model adjusted for age group, gender, and race, and bars indicate 1 standard error.

We first examined the effect of immunosuppression on surface expression of TLRs 1, 2, and 4 as these TLRs are among those crucial for initiating an innate immune response against most microbial pathogens and some viruses. We labeled TLRs on living PBMCs on the day of isolation and quantified the % positive cells by flow cytometry for subjects from each group. Previously we have used this method to demonstrate an age-dependent decrease in expression of TLR1 but not TLR2 on monocytes [13]. There was no significant difference in mean percentage positive monocyte surface expression of TLR1 or TLR2 in non-immunosuppressed adults (n = 419) compared to immunosuppressed adults (n = 137) (Fig. 1; mean percentage positiveTLR1 60.09±2.63 S.E. versus 60.59±3.38 S.E., p = 0.90; TLR2 90.56±0.52S.E. versus 91.59±0.4S.E., p = 0.07). In contrast, mean percentage positive monocyte surface expression of TLR4 was significantly increased in immunosuppressed adults (Fig. 1; mean percentage positive 70.12±2.28 S.E.versus 61.72±2.05 S.E., p = 0.0008). We have previously reported that TLR4, which recognizes LPS, is expressed at lower levels on the surface of monocytes from older compared to younger adults [13]. The increase in immunosuppressed adults was primarily observed in the youngest and oldest age groups (age≤40 and ≥60, p =  0.0011 and 0.0004 respectively; age 40-59, p = 0.71, data not shown). To assess the consistency of these findings within this broadly defined cohort of immunosuppressed adults we conducted subset analyses to quantify differences in TLR expression for RA adults only, SLE adults only or “other” immunosuppressed adults compared to non-immunosuppressed subjects. Within these disease groups, TLR4 surface expression remained significantly increased compared to cells from non-immunosuppressed adults (data not shown).

### TLR-induced cytokines are increased in immunosuppressed adults

**Figure 2:**
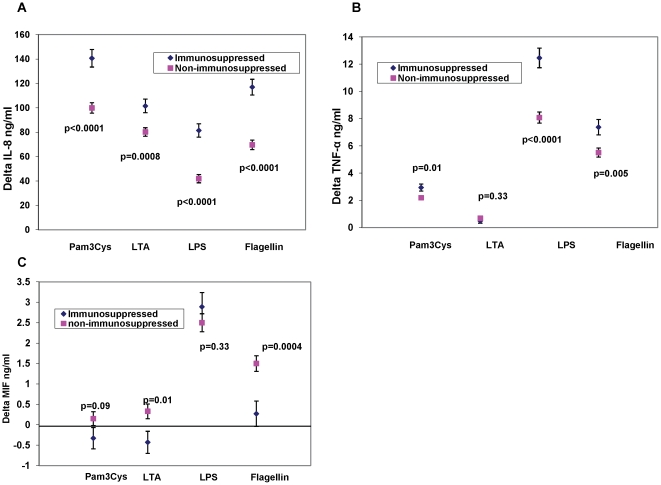
TLR signaling efficiency in Immunosuppressed adults compared to Non-immunosuppressed adults. Delta IL-8 (Panel A) and Delta TNFα (Panel B) and Delta MIF (Panel C) levels in monocytes (Delta  =  units changed from the baseline unstimulated levels.) TLR ligands were as follows: For TLR1/2, Pam_3_CSK_4_; 5 µg/ml; for TLR2, LTA; 1 µg/ml; for TLR4, LPS: 0.5 µg/ml, for TLR5 flagellin 2.5 µg/ml. Values indicate least squares means from a model adjusted for age group, gender, and race, and bars indicate 1 standard error.

We have shown previously that reduced expression of certain TLRs in samples from older compared to younger adults is associated with reduced production of inflammatory cytokines after TLR ligand stimulation [13], [15]. We next assessed whether the elevated levels of TLR4 noted in immunosuppressed adults were associated with functional changes in cytokine production in this population. Adherent monocytes were stimulated *in vitro* for responsiveness to TLR ligands including those for the TLR 1/2 heterodimer (Pam3CSK4), the TLR 2/6 heterodimer (LTA), TLR4 (LPS) and TLR5 (flagellin), and the production of cytokines was quantified by ELISA. In particular, we examined levels of IL-8 and TNFα, which are among the first cytokines to be secreted by macrophages particularly upon infection with certain viruses [20], [21]. After adjusting for covariates of age, gender and race, we found that levels of IL-8 produced by monocytes from immunosuppressed adults were significantly higher after stimulation with TLR-specific ligands than levels from non-immunosuppressed adults (Figure 2A: Pam_3_Cys p = <0.0001, LTA p = 0.0008, LPS p<0.0001, flagellin p<0.0001). Similarly, levels of TNF-α produced by cells from immunosuppressed adults were elevated after stimulation with ligands for the TLR 1/2 heterodimer, TLR4, and TLR5 (Fig. 2B). Basal production (absolute amount of a given cytokine measured prior to addition of any ligand) of IL-8 and TNF-α was similar in the immunosuppressed adults compared to non-immunosuppressed subjects (Mean IL-8 basal levels:Immunosuppressed: 35.97 ng/dl (5.07 S.E.), non-immunosuppressed: 39.92 (3.32 S.E.) p =  0.48; Mean TNF basal levels:Immunosuppressed: 0.48 ng/dl (0.11 S.E.),non-immunosuppressed: 0.33 (0.07 S.E.), p =  0.22).

In addition to IL-8 and TNFα, we quantified levels of MIF, a protean pro-inflammatory mediator whose expression is increased in patients with RA and has been associated with severity of several autoimmune conditions [22]-[26]. Baseline levels of MIF were constitutively higher in the immunosuppressed compared to the non-immunosuppressed cohort (p<0.0001 in unstimulated cells). After adjusting for baseline differences, MIF levels in immunosuppressed adults relative to those from cells of non-immunosuppressed adults after stimulation with LTA and flagellin were significant lower (Fig. 2C, p = 0.01 and p = 0.0004; respectively). These results were again consistent when the immunosuppressed cohort was analyzed within the three subsets; that is, adults with only RA, only SLE or only “other disease” relative to non-immunosuppressed adults showed similar findings (results not shown).

**Figure 3:**
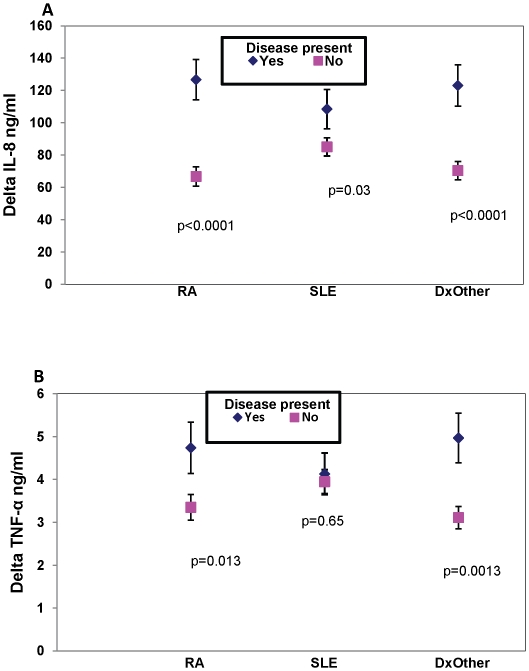
Effect of underlying disease on cytokine production. When compared to adults who did not have the diagnosis of RA, SLE or “Diagnosis Other” (all other diseases for which adults were taking immunosuppressant medication), those with RA or “diagnosis other” had significantly higher IL-8 (3A) and TNFα (3B) levels. MIF levels were not significantly associated with the diagnosis of an underlying autoimmune disease (not shown). Values indicate least squares means from a model adjusted for age group, gender, and race and bars indicate 1 standard error.

As shown in Table 2, our recruited immunosuppressed adults include those with several autoimmune conditions who are taking biologic, non-biologic, or both classes or medication. A multivariable analysis was performed to assess the effect of underlying disease and medication class on measured cytokine levels. Levels of IL-8 were higher among those with specific autoimmune disease relative to those without (Fig. 3A). For TNFα, those with RA and other immunosuppressed diseases had higher levels however this was not observed in adults with SLE (Fig. 3B). This may reflect a signaling process that is undefined but unique in SLE. In contrast to IL-8 and TNFα, MIF levels were not significantly affected by underlying diagnoses (data not shown).

Medication class was not associated with TNFα levels (overall p = NS; data not shown). The overall effect of medication class on IL-8 levels was also not significant, however, pairwise comparisons showed that IL-8 levels were reduced by 30.0±13.9 units (mean±S.E.) for biologics (n = 22) compared to no medication (n =  432) (p = 0.031) and 27.9±12.6 units (mean±S.E.) for non-biologics (n =  69) compared to no medication (p = 0.027). Absolute MIF levels were associated with medication class (overall effect of medication p = 0.038).

## Discussion

Adults with autoimmune disease on immunosuppressive regimens have higher morbidity and mortality rates due to infection. These increases may be due to both aberrant and impaired immune response as well as immunosuppressive drugs that induce cytokine blockade or impaired call activation. The degree to which the underlying autoimmune disease increases this risk relative to that conferred by immunosuppressive medications is unknown. Current evidence suggests that there are aspects of both the underlying disease and medications (especially corticosteroids and cyclophosphamide) that increase infectious complications in SLE (reviewed in 27). Similar findings also suggest an independent risk for infection in patients with rheumatoid arthritis, rheumatoid factor positivity, and glucocorticoid use based on a recent nested cohort study of a large group of patients with inflammatory arthritis in the UK [28]. Our findings provide a description of the innate immune responses in a closely followed cohort of immunosuppressed adults. While the sample population is heterogeneous in terms of disease diagnoses and severity, these adults often present for clinical care with multiple factors contributing to an immunosuppressed state: alterations in immune response may reflect underlying disease, immunosuppressive medication regimen, or both. It is for these reasons we found it useful to broadly examine the initial innate immune response mediated by TLRs in this context.

We have shown that monocytes from immunosuppressed adults have a significant increase in surface expression of TLR4, produce high levels of the proinflammatory cytokines IL-8 and TNFα in vitro in response to stimulation of TLR1/2, TLR2/6, TLR4 and TLR5 (despite similar baseline levels) and constitutively produce higher levels of the proinflammatory cytokine MIF compared to non-immunosuppressed adults. We observed these differences in cytokine production in vitro not only in the heterogeneous group of immunosuppressed subjects as a whole, but also in subsets restricted to individuals with RA, SLE or “other” disease states. The explanation for the broad elevation of cytokine production in cells from immunosuppressed adults is likely multifactorial; we found increased levels of TLR4 protein on the surface of monocytes from immunosuppressed compared to non-immunosuppressed individuals a potential contributor to increase cytokine production in response to LPS stimulation. However, levels of TLRs 1 and 2 appear unperturbed on the surface of monocytes in immunosuppressed and non-immunosuppressed individuals despite differences in cytokine production after stimulation, suggesting that alterations in TLR intracellular signaling related to the underlying disease processes are also likely. In view of previously published work suggesting a role for MIF in the expression level of TLR4 [29], [30], these results suggest an association between TLR4 and MIF in patients with autoimmune disease.

This link between increased TLR4 surface expression and MIF cytokine production is compelling since several lines of evidence are emerging that suggest a regulatory relationship between the two. The TLR4 agonist LPS is a known stimulus for MIF production by monocytes [31]. Furthermore, Roger and colleagues found that mouse macrophages genetically deficient in MIF expressed significantly lower levels of TLR4 protein [30]. Our results are consistent with the notion that MIF up-regulates TLR4 expression and that TLR4 stimulation in turn leads to the production of MIF, thus establishing a mutually-reinforcing, pro-inflammatory feedback mechanism [29].

A sizeable body of work has emerged over the past 5 years elucidating the role of MIF in the pathogenesis of autoimmune disease including RA. Proposed mechanisms by which MIF may play a role in the joint destruction seen in RA include up-regulation of metalloproteinase expression, promotion of IL-1-induced inflammatory cascades, and reduction of synovial fibroblast apoptosis via MIF-induced inhibition of p53 [32]-[34]. In patients with RA, high concentrations of MIF have been found in the serum and synovial fluid and high levels of circulating MIF correlate with joint damage [26], [35]. High serum MIF levels were associated with polymorphisms in a functional promoter region of the *MIF* gene and patients with high-risk *MIF* alleles (-173C or CATT7) had higher serum MIF levels that were associated with erosive changes over a 6 year follow-up. We found that elevated MIF levels are significantly associated with immunosuppressive medication use whereas IL-8 and TNFα showed less or no overall association, respectively. While MIF is associated with use of immunosuppressive medication, which may be related to the underlying severity of disease, we are unable to make a more firm conclusion about this since formal measures of disease severity were not available in our studied cohort. Further research to define the precise effect(s) of immunosuppressive medication on MIF production is warranted. In addition, the specific correlation between functional MIF gene polymorphisms and TLR4 expression in healthy and immunosuppressed patients is worthy of further investigation.

A limitation of the current study is the heterogeneous nature of the immunosuppressed subject's disease states and medications. In this regard, the subset analyses we carried out in which the primary findings of increased TLR4 surface expression, high proinflammatory cytokine levels after stimulation by most TLR ligands and higher constitutive MIF levels were observed in all three autoimmune disease groups as well as the immunosuppressed group as a whole indicates that these results were not driven by one subset of immunosuppressed patients. Another potential limitation would be a residual effect of immunosuppressive medication on the *in vitro* assays of TLR function, although we took measures in our experimental procedures to mitigate medication effects (i.e. overnight incubation, cell washing, etc.). Taken together, our results provide strong evidence for dysregulation of the innate immune system in the context of both autoimmune disease and immunosuppression medication use. Future studies are needed to elucidate the contribution of these differences to outcome from infection or on the progression of underlying autoimmunity.

## Materials and Methods

### Study Participants

This study was approved by the Human Investigations Committee at the Yale University School of Medicine. Written informed consent was obtained from all volunteers.

Immunosuppressed adults over the age of 21 were recruited from outpatient rheumatology clinics in the greater New Haven, Connecticut area. Patients were enrolled between October, 2005 and March, 2009. Immunosuppressed subjects were defined as those taking at least 10 mg of prednisone daily or other biologic and/or non-biologic immunosuppressive medications (for a complete list of medications represented see Table 2). The majority of subjects had rheumatoid arthritis (RA), systemic lupus erythematosis (SLE), or polymyalgia rheumatica (PMR); diagnoses were in accord with criteria defined by the American College of Rheumatology. Patients were excluded if they were non-ambulatory or in a nursing home, pregnant, treated for cancer in the previous 3 months, the recipient of a bone marrow or solid organ transplant, or had taken antibiotics or reported a fever within the two weeks prior to enrollment. Non-immunosuppressed adults were enrolled from Yale Health Services and were part of a previously described cohort [13]. Subjects in either group were not excluded if they had a chronic medical condition such as diabetes or high blood pressure although these other medical conditions occurred at an estimated frequency of less than 5%.

### Isolation and labeling of peripheral blood mononuclear cells and flow cytometery

Peripheral blood mononuclear cells (PBMC) were isolated from heparinized blood by Ficoll-Hypaque density gradient centrifugation as previously described [13]. Surface expression of TLRs 1, 2, and 4 was assessed on living cells on the day of isolation. Cell suspensions were stained in PBS with 1% FBS for 30 min on ice (protected from light) with the following antibodies: CD4 (PE-Cy5, clone RPA-T4), TLR1 (PE, clone GD2.F4), TLR2 (FITC clone TLR2.1), TLR4 (PE, clone HTA 125). Cells were washed, resuspended in 1% paraformaldehyde in PBS, and stored in the dark at 4°C until assessment by FACS within 24 hours. Data were acquired on a FACS Calibur instrument (BD Biosciences) and analyzed using FlowJo software (Tree Star). We acquired 40,000 events per sample, and monocytes were gated as CD4-dim cells as previously described [14]. All antibodies were purchased from eBioscience.

### Cell Stimulation

For assessment of TLR signaling efficiency, PBMC were plated in 48 well plates (BD-Falcon) at 5×10^5^/well in RPMI 1640 medium containing 20% human serum (Lonza, MD), 1000 U/ml penicillin, and 1000 µg/ml streptomycin (InvivoGen, Carslbad, CA) as described [15]. After 2 hr, non-adherent cells were washed away and cells were stimulated for 20 hr with TLR ligands as follows: For TLR1/2, N-palmitoyl-S-[2,3-bis(palmitoyloxy)-(2R,S)-propyl]-Cys-[S]-Serl-[S]-Lys[4] trihydrochloride (Pam_3_CSK_4_; 5 µg/ml); for TLR2, lipoteichoic acid (LTA; 1 µg/ml); for TLR4, lipopolysaccharide (LPS: 0.5 µg/ml, Sigma, St. Louis, MO); for TLR5 flagellin (2.5 µg/ml). Medium alone served as a control. All ligands were obtained from InvivoGen (Carlsbad, CA) except as noted. Culture supernatants from adherent cells were harvested and stored at -80°C until use. Production of interleukin 8 (IL-8) and tumor necrosis factor α (TNFα) was quantified by enzyme-linked immunosorbent assays (ELISAs) using cytokine-specific capture antibodies, biotinylated monoclonal detection antibodies, and recombinant human cytokine standards according to the manufacturer's instructions (BD PharMingen, CA). Levels of macrophage migration inhibitory factor (MIF) were measured by ELISA as described previously [16]. The cytokine level in each sample was determined twice.

### Statistics

Proportions were used to describe the demographic and clinical characteristics of each cohort at enrollment. We estimated the association between immunosuppression status and TLR surface expression for TLRs 1, 2, and 4 in the cohort using generalized linear models controlling confounding variables by including the covariates age group (21-39, 40-59, ≥60), gender, race, and year sampled [17].

In order to model both the variation in our sample of immunosuppressed and non-immunosuppressed adults, as well as the correlation between ligand specific stimulation, and the interaction between immunosuppression status and ligands, we employed a mixed effects model to estimate the effect of immunosuppression on IL-8, TNF-α and MIF percentage change after stimulation in PMBCs [18], [19]. Specifically, we used an unstructured covariance structure that permitted each participant to have a unique correlation structure for each ligand stimulation; this accounted for the inherent variation of each participant. The same covariates were included to control for confounding. Least squares means were estimated for the fixed effects of immunosuppression status by ligand interaction and the differences were tested.

Subsequently, we used mixed effects models to estimate the association between RA, SLE, and other immunosuppressive diseases with the percentage change in IL-8, TNF-α and MIF production after stimulation of PBMCs. In the same models we estimated the association with medications for autoimmune diseases adjusting for correlations between ligand specific stimulation and covariates listed above.

Statistical tests were 2-tailed, and p<0.05 considered to indicate statistical significance. Analyses used SAS version 9.2 (SAS Institute, Cary, NC).
